# Comparative transcriptomics of choroid plexus in Alzheimer’s disease, frontotemporal dementia and Huntington’s disease: implications for CSF homeostasis

**DOI:** 10.1186/s12987-018-0102-9

**Published:** 2018-05-31

**Authors:** Edward G. Stopa, Keith Q. Tanis, Miles C. Miller, Elena V. Nikonova, Alexei A. Podtelezhnikov, Eva M. Finney, David J. Stone, Luiz M. Camargo, Lisan Parker, Ajay Verma, Andrew Baird, John E. Donahue, Tara Torabi, Brian P. Eliceiri, Gerald D. Silverberg, Conrad E. Johanson

**Affiliations:** 1 Departments of Neurosurgery and Pathology (Neuropathology Division), Rhode Island Hospital, The Warren Alpert Medical School, Brown University, Providence, RI USA; 2 Genetics and Pharmacogenomics, Merck & Co., Inc., West Point, PA USA; 3 United Neuroscience, Dublin, Ireland; 4 Department of Surgery, University of California San Diego Medical Center, Hillcrest, 212 Dickinson Street, San Diego, CA USA

**Keywords:** Choroid plexus transcriptome, Neuroimmune CSF regulation, Blood–CSF barrier inflammatome, Janus kinase/signal transducers and activators of transcription (JAK-STAT), Peroxisome-proliferator-activated receptor (PPAR), Cadherin-mediated adhesion, Vascular endothelial growth factor, LRP-1, Choroid plexus methionine, CSF homocysteine, Mechanistic target of rapamycin (mTOR)

## Abstract

**Background:**

In Alzheimer’s disease, there are striking changes in CSF composition that relate to altered choroid plexus (CP) function. Studying CP tissue gene expression at the blood–cerebrospinal fluid barrier could provide further insight into the epithelial and stromal responses to neurodegenerative disease states.

**Methods:**

Transcriptome-wide Affymetrix microarrays were used to determine disease-related changes in gene expression in human CP. RNA from post-mortem samples of the entire lateral ventricular choroid plexus was extracted from 6 healthy controls (Ctrl), 7 patients with advanced (Braak and Braak stage III–VI) Alzheimer’s disease (AD), 4 with frontotemporal dementia (FTD) and 3 with Huntington’s disease (HuD). Statistics and agglomerative clustering were accomplished with MathWorks, MatLab; and gene set annotations by comparing input sets to GeneGo (http://www.genego.com) and Ingenuity (http://www.ingenuity.com) pathway sets. Bonferroni-corrected hypergeometric p-values of < 0.1 were considered a significant overlap between sets.

**Results:**

Pronounced differences in gene expression occurred in CP of advanced AD patients vs. Ctrls. Metabolic and immune-related pathways including acute phase response, cytokine, cell adhesion, interferons, and JAK-STAT as well as mTOR were significantly enriched among the genes upregulated. Methionine degradation, claudin-5 and protein translation genes were downregulated. Many gene expression changes in AD patients were observed in FTD and HuD (e.g., claudin-5, tight junction downregulation), but there were significant differences between the disease groups. In AD and HuD (but not FTD), several neuroimmune-modulating interferons were significantly enriched (e.g., in AD: IFI-TM1, IFN-AR1, IFN-AR2, and IFN-GR2). AD-associated expression changes, but not those in HuD and FTD, were enriched for upregulation of VEGF signaling and immune response proteins, e.g., interleukins. HuD and FTD patients distinctively displayed upregulated cadherin-mediated adhesion.

**Conclusions:**

Our transcript data for human CP tissue provides genomic and mechanistic insight for differential expression in AD vs. FTD vs. HuD for stromal as well as epithelial components. These choroidal transcriptome characterizations elucidate immune activation, tissue functional resiliency, and CSF metabolic homeostasis. The BCSFB undergoes harmful, but also important functional and adaptive changes in neurodegenerative diseases; accordingly, the enriched JAK-STAT and mTOR pathways, respectively, likely help the CP in adaptive transcription and epithelial repair and/or replacement when harmed by neurodegeneration pathophysiology. We anticipate that these precise CP translational data will facilitate pharmacologic/transgenic therapies to alleviate dementia.

**Electronic supplementary material:**

The online version of this article (10.1186/s12987-018-0102-9) contains supplementary material, which is available to authorized users.

## Background

The choroid plexus (CP) is a CNS secretory tissue within the cerebroventricular system consisting of a vascular stroma surrounded by epithelium [1, 2]. Although the primary function of choroidal tissue is to produce and regulate cerebrospinal fluid (CSF), it also importantly provides a permeability-regulating blood–CSF barrier (BCSFB) [3]. Other additional roles of CP relate to CNS wound repair [4], sex hormone modulation of BCSFB-CNS [5], catabolite detoxification [6], ion regulation [7], a selective leukocyte gate [8], and CSF–brain neuroimmune homeostasis, including interferon actions [9–13]. Recently, the BCSFB tissue has been examined for unique CP changes in diverse disorders: mitochondrial diseases [14], multiple sclerosis/experimental autoimmune encephalitis [15, 16], schizophrenia [17], acute response to peripheral immune challenge [18], normal pressure hydrocephalus [19], and Alzheimer’s disease (AD) [20, 21].

Analyzing the transformed CP tissue composition and pathophysiologic functions in neurodegeneration elucidates specific metabolic/secretory processes underlying CSF–CNS disease pathogenesis [22]. BCSFB alterations such as choroid epithelial cell atrophy, stromal fibrosis, vascular thickening, tight junction (claudin-5) downregulation, and basement membrane thickening are associated with AD pathology [20]. These changes in the epithelial–stromal nexus likely affect secretion and transport, resulting in diminished CSF turnover and modified neuroimmune regulation. Neuroimmune phenomena in the CP and/or CSF include adjustments in the level of proteins (e.g., neurotrophins, interferons and growth factors), cytokines, and certain immune cells [12, 22]. Oxidative stressors in AD and other dementias may also differentially impact CP’s ability to synthesize/transport proteins/hormones, and to regulate cellular/CSF metabolites such as methionine/homocysteine [23], Aβ/tau [24], and creatine/creatinine [25].

This investigation at the Brown University Medical School, in collaboration with Merck & Co., analyzed gene expression in CP tissues from late-stage Alzheimer patients, for comparison with control subjects (Ctrl) and two other diseases: frontotemporal dementia (FTD) and Huntington’s disease (HuD). Our working hypothesis anticipated: (i) common denominators of altered CP expression in the three diseases, as well as (ii) differential expression patterns due to disease-specific alterations in neural metabolites, that by ‘homeostatic feedback signaling’ via volume transmission from brain to CSF to CP, could uniquely modulate gene expression at the BCSFB.

Investigating CP tissue gene expression in various CNS diseases likely informs on diverse BCSFB adjustments to neurodegeneration. Bergen et al. [26] focused on gene expression changes by CP epithelial cells in AD. In this study, we analyze CP tissue responses (epithelium plus stroma) by providing profiles of mRNA changes. The altered expression profiles in AD, FTD and HuD are discussed in relation to restorative homeostatic mechanisms, as well as to chronic BCSFB damage and disrupted CSF–brain homeostasis.

## Methods

### Project approval, sample collection and demographics

This research with banked specimens of human CP tissue was approved by the Institutional Review Board for Clinical Research at *Lifespan*, Rhode Island Hospital, Providence, RI. Post-mortem tissue samples from 6 healthy Ctrls of mean age 60 years, mean post-mortem interval (PMI) 22 h; and from 7 patients with advanced AD (Braak and Braak stage III–VI, 80 years, PMI 17 h), 4 FTD (72 years, PMI NA) and 3 HuD (71 years, PMI 19 h), were snap frozen in liquid N_2_ and stored at − 80 °C in the Brown University Brain Tissue for Neurodegenerative Disorders Resource Center until processed. Demographic and disease data for the individual controls and patients are presented in Table 1.

**Table 1:** Demographic and clinical data of choroid plexus samples collected

Sample ID #	Diagnosis	Age	Sex	PMI
CP_CTR_007	Control	62	M	23.9
CP_CTR_008	Control	55	F	29
CP_CTR_009	Control	37	M	18.7
CP_CTR_010	Control	64	M	25.8
CP_CTR_011	Control	69	F	12.3
CP_CTR_012	Control	70	M	N/A
CP_ALZ_015	AD (Braak III–IV)	74	F	15
CP_ALZ_017	AD (severe Braak V–VI)	84	F	N/A
CP_ALZ_018	AD (severe Braak V–VI)	84	M	N/A
CP_ALZ_019	AD (severe + Lewy body disease)	84	F	N/A
CP_ALZ_020	AD (severe Braak V–VI)	89	M	N/A
CP_ALZ_022	AD (severe Braak V–VI)	73	M	24.5
CP_ALZ_023	AD (severe Braak V–VI)	70	M	10.8
CP_FTD_024	FTD	76	M	N/A
CP_FTD_025	FTD and motor neuron disease	75	F	N/A
CP_FTD_026	FTD Pick’s disease	58	M	N/A
CP_FTD_027	FTD	80	F	N/A
CP_HuD_029	HuD (grade IV)	68	M	30.1
CP_HuD_030	HuD (grade IV)	65	F	3.5
CP_HuD_031	HuD (grade IV)	80	M	24

### Microarray design and analysis

RNA from CP was extracted using TRIzol reagent in accordance with instructions by manufacturer (Thermo-Fisher, Grand Island, NY). Isolated RNA samples were assayed for quality via the Agilent RNA 6000 Pico Kit on Agilent Bioanalyzer (Santa Clara, CA) and RNA yield via Quanti-iT RiboGreen RNA Assay Kit (Thermo-Fisher). Samples were amplified and labeled using an automated version of the NuGEN Ovation WB protocol after normalizing to 50 ng total RNA input (NuGEN Technologies, San Carlos, CA). Gene expression profiling was performed with a customized Human Affymetrix GeneChip microarray (GEO platform GPL 10379) that included 57,060 probe sets (Affymetrix, Santa Clara, CA). Hybridization (45 °C for 18 h), labeling, and scanning, using Affymetrix ovens, fluidics stations, and scanners, were conducted following the protocols recommended (NuGEN Technologies). All 20 samples passed RNA integrity and Affymetrix quality control metrics. The final sample set contained RNA from 6 Ctrl, 7 AD, 4 FTD and 3 HuD subjects (Table 1, Additional file 1: Table S1).

### Data processing, statistics and annotation

Data were normalized by robust multiarray average (RMA) [27], and each sample was ratioed to the average of the Ctrl samples [28]. Statistical analysis and agglomerative clustering were performed using MathWorks MatLab (Natick, MA). In some statistical analyses, due to insufficient power, HuD and FTD data were combined into one non-AD disease grouping, as indicated by: (HuD + FTD). Gene set annotation analysis was performed by comparing input sets to GeneGo (http://www.genego.com) and Ingenuity (http://www.ingenuity.com) pathway sets. Bonferroni-corrected hypergeometric p-values (expectation (e)-values) of <0.1 were considered a significant overlap between sets.

## Results

**Fig. 1:**
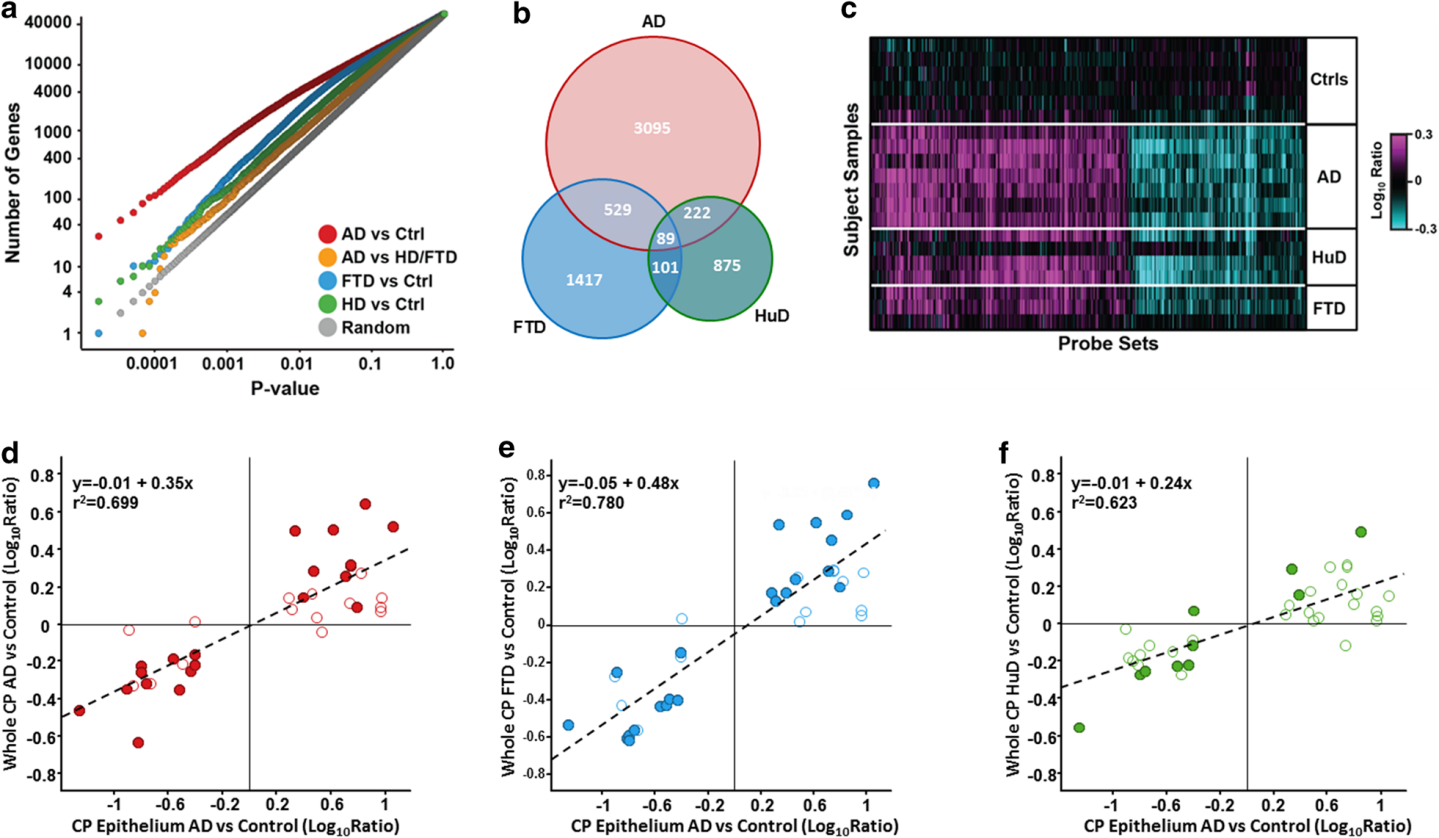
Significant gene expression differences between Ctrl and diseased CP: **a** T-test p-value distributions among all probe sets for AD (red), FTD (blue) and HuD (green) vs. Ctrl samples, as well as AD vs. combined FTD plus HuD samples (orange). Gray data points indicate number of significant probe sets expected by chance. **b** Overlap of probe sets differentially expressed (p < 0.01) between Ctrl and AD (red), Ctrl and FTD (blue) and Ctrl and HuD (green) subjects. **c** Heatmap of probe sets differentially expressed (p < 0.01) between AD and Ctrl subjects. Probe sets were ordered by agglomerative clustering. Correlation between expression changes in whole CP from AD (**d**), FTD (**e**), and HuD (**f**) to those reported by Bergen et al. [26] in laser-dissected CP epithelial cells from AD subjects. Plotted are the 36 genes reported in Tables 1 and 2 by Bergen et al. [26] that were also represented on the array used in our study. Values are relative to corresponding study Ctrl subjects. Filled circles had p < 0.05 in the corresponding whole CP comparisons. Dotted lines, the provided equation and r^2^ values represent linear fit of the data

We first compared the genome-wide differences in gene expression between diseased and Ctrl CP. p-value distributions from T-test comparisons, between the Ctrl group and each of the neurodegenerative disease groups (AD, HuD, FTD), revealed significant effects on the CP transcriptome in each of the 3 diseases (Fig. 1a, Additional file 2: Table S2). The AD group had the highest number of differentially expressed probe sets likely due, at least in part, to the higher number of AD subjects compared to HuD and FTD. 3935 (7%) out of the 57,060 probe sets on the array were differentially expressed [p < 0.01, false discovery rate (FDR) = 14%] between AD and Ctrl subjects, while 1287 (FDR = 44%) and 2136 (FDR = 27%) probe sets were regulated with p < 0.01 in HuD and FTD, respectively (Fig. 1a, b, Additional file 2: Table S2). Despite the limited statistical power and resulting high false discovery rates in the HuD and FTD comparisons, there was a large degree of overlap in the genes identified in each of the comparisons (Fig. 1b, c, Additional file 2: Table S2). Almost all probe sets differentially expressed (p < 0.01) in the AD samples had significant or a trend toward differential expression in the same direction in the other two disease groups (Fig. 1c, Additional file 2: Table S2). In Additional file 4: Table S4 are listed the top 10 most upregulated and 10 most downregulated genes from the AD vs. Ctrl comparison; 80% of these findings were confirmed by multiple probe sets when available on the array.

In order to validate these findings with different subjects and gene expression platforms, we compared the results in whole CP to those obtained by Bergen et al. in laser-dissected CP epithelial cells from control and AD subjects [26]. We anticipated that the changes reported in CP epithelial cells would also be evident in the whole CP samples but to a lesser degree given the presence of additional cell types such as stroma and immune cells in the whole tissue samples. Indeed, the 36 genes reported as differentially expressed in AD CP epithelium by microarray, and in some cases [17] also by quantitative PCR (Bergen et al. Tables 1 and 2 in [26]), were regulated similarly in AD whole CP tissue in our study (r^2^ ~ 0.7), but to a lesser magnitude (by ~ 35%, as indicated by linear regression in Fig. 1d). 34 of the 36 differentially expressed genes reported by Bergen et al. [26] were modulated in the same direction from Ctrl in both studies, with 20 obtaining significance (p < 0.05) in the current AD whole CP tissue comparison. Similarly the expression values in whole CP from FTD and HuD also correlated with those reported for AD CP epithelium in reference # [26] (r^2^ = 0.8 and 0.6, respectively, Fig. 1e, f).

**Fig. 2:**
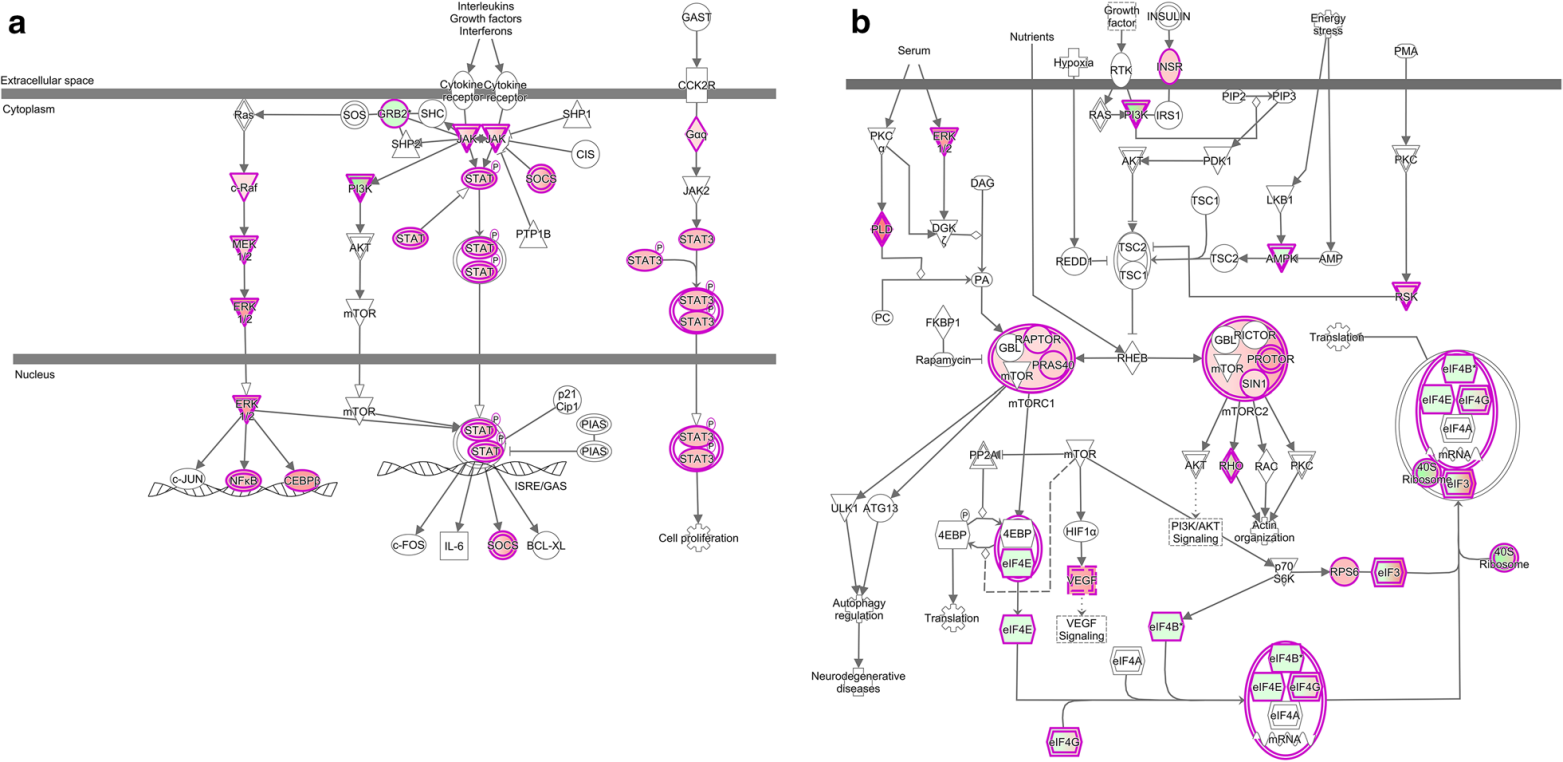
Up-regulation of the JAK-STAT and mTOR pathways: Ingenuity pathway maps for **a** JAK-STAT signaling (transducing extracellular signals to transcriptional responses) and **b** mTOR signaling (a master regulator for many fundamental cellular repair processes). Genes with AD vs. Ctrl (p < 0.01) are outlined in red, and filled with red or green, indicating the magnitude of increased or decreased expression, respectively, in AD

In this study, among the 3935 probe sets differentially expressed (p < 0.01) in AD compared to Ctrl subjects, 2332 were upregulated and 1603 downregulated (Fig. 1b, Additional file 2: Table S2). The differentially expressed genes were examined for overlap with ~ 2000 GeneGo and Ingenuity pathways. Ninety-two pathways were enriched (Bonferroni corrected p-value, i.e., e-value, < 0.01) among the upregulated genes. These enrichments represented primarily immune-related pathways, including acute phase response, cytokine and interferon signaling, NFkB, and cell adhesion, as well as growth factor, JAK-STAT and mTOR signaling pathways, PPAR signaling and protein/nucleic acid salvage pathways (Fig. 2, Additional file 3: Table S3a). Pathway enrichment among downregulated genes was less extensive (12 pathways with e < 0.01), including genes involved in methionine degradation and protein translation (Fig. 2, Additional file 3: Table S3b).

**Fig. 3:**
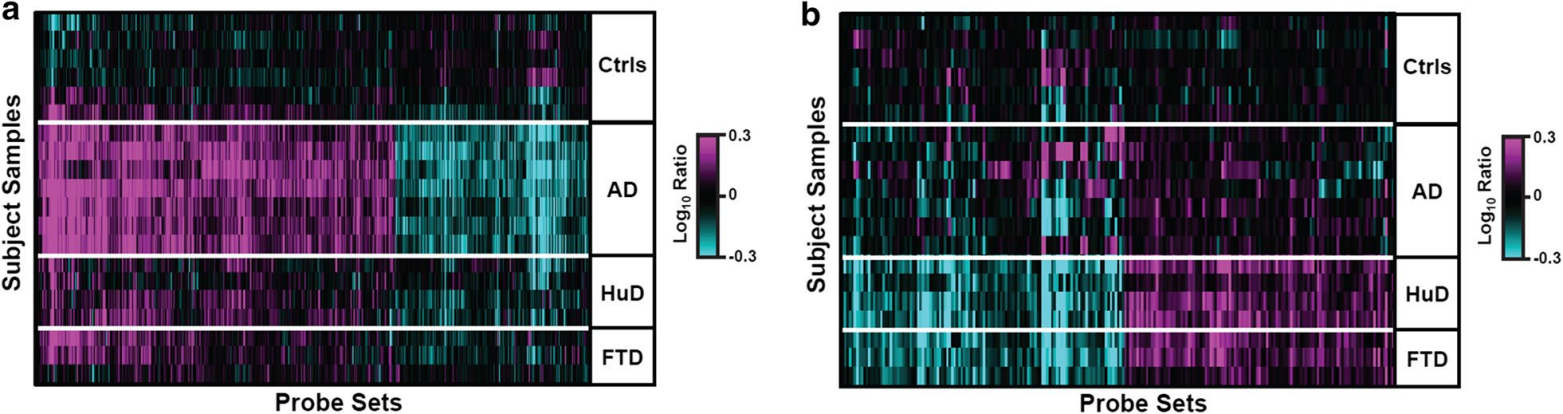
Expression changes unique to AD, or to the combined HuD + FTD, non-AD ‘disease control’ group: **a** Heatmap of probe sets differentially expressed between AD and Ctrl subjects (p < 0.01), and altered more relative to the control in the AD group than in the non-AD ‘disease control’ group (AD/Ctrl)/(HuD + FTD/Ctrl) > 1, and AD vs. HuD + FTD, (p < 0.05). Probe sets are ordered by agglomerative clustering. **b** Heatmap of probe sets differentially expressed between the combined HuD + FTD group and Ctrl subjects (p < 0.01), and altered more relative to the combined HuD + FTD disease control group than in the AD group (HuD + FTD/Ctrl)/(AD/Ctrl) > 1, and HuD + FTD vs. AD, (p < 0.05). Probe sets were ordered by agglomerative clustering. Red (magenta) and green (cyan) indicate the magnitude of increased and decreased expression, respectively

Many differences between AD and Ctrl were also observed in HuD vs. FTD (Fig. 1, Additional file 2: Table S2). However, there were also some significant differences between AD vs. HuD + FTD, with 902 (1.6%) probe sets significant at p < 0.01 (63% FDR) (Fig. 3). Figure 3a displays the genes up and down-regulated in AD more than in the combined HuD + FTD group; whereas Fig. 3b presents the opposite, i.e., genes regulated more in HuD + FTD than in AD. The 513 probe sets uniquely upregulated in AD: AD vs. Ctrl (p < 0.01) and AD vs. HuD + FTD (p < 0.05) were enriched (e < 0.1) predominately in interleukin and VEGF signaling genes (Additional file 3: Table S3c). There were 272 probe sets uniquely downregulated in AD: AD vs. Ctrl (p < 0.01) and AD vs. HuD + FTD (p < 0.05) but were not significantly (e < 0.1) enriched in any queried pathway. The 112 probe sets uniquely upregulated in HuD + FTD, that is, HuD + FTD vs. Ctrl (p < 0.01), and HuD + FTD vs. AD (p < 0.05), were enriched (e < 0.1) in cadherin-mediated cell adhesion (Additional file 3: Table S3d). The 115 probe sets uniquely downregulated in AD vs. Ctrl (p < 0.01), and AD vs. HuD + FTD (p < 0.05), were not significantly (e < 0.1) enriched in assessed biological pathways.

The emphasis here is on gene sets and associated biological pathways. Still, it is instructive to focus on several genes/proteins currently of great interest in CP pathophysiology. Altered tight junction protein claudin-5 in CP is associated with BCSFB breaching [26], while interferon has a protein-signaling role that couples choroidal-cerebral neuroimmune interactions [13]. Claudin-5 was downregulated 1.6-, 1.9- and 2.5-fold in AD, FTD and HuD, respectively, reaching significance in FTD (respective p values of 0.071, 0.028 and 0.059). Multiple interferon signaling genes were upregulated in AD: IFI-TM1 (p = 0.0008), IFN-AR1 (p = 0.006), IFN-AR2 (p = 0.0007) and IFN-GR2 (p = 0.0002). The complete lists of differentially-expressed genes, as well as enriched biological pathways, are provided in Additional file 2: Table S2, Additional file 3: Table S3, Additional file 4: Table S4.

## Discussion

Comparing gene expression in CP of AD subjects to that of Ctrls and other neurodegenerative diseases reveals important biological functions altered by dementia generation and progression. The CP as a dynamic interface between blood, CSF and brain, is able to monitor distortions and homeostatically respond, e.g., by the JAK-STAT pathway as well as cytokine and protein-signaling molecules [12, 13]. These homeostatic adjustments impact neural viability. This intimates that CP beneficial adjustments, as well as BCSFB malfunctioning, are pertinent to AD progression. The strategic role of CSF to safeguard brain is manifested by CP upregulation of many genes in response to neurodegeneration ([26] and this investigation); and by its ability to protect basic CSF composition (e.g., K, pH and vitamin homeostasis) even in advanced AD [7]. Nevertheless, the CP in AD incurs structural damage [20] and distorted epithelial metabolism and transport (e.g., Aβ, cytokine (e.g., TNFα) and methionine/homocysteine [24, 29]). The injured epithelium likely depends on enhanced mTOR expression to facilitate cellular repair and/or replacement. Accordingly, the state of CP–CSF viability in ongoing neurodegeneration is a balance between debilitating and restorative events at the BCSFB [29–31].

In this study we have shown numerous transcriptional alterations in the CP of subjects with neurodegenerative disease. Given that the BCSFB makes adjustments in CSF composition, the prolific disease-induced expression changes in CP fits previous homeostasis modeling [1, 30]. Most transcript changes were common to AD, FTD and HuD, while fewer genes were modulated differently between AD and HuD + FTD. It is instructive to compare the present observations with an earlier transcriptional investigation of the BCSFB in AD [26]. Whereas we studied homogenates of entire CP, including epithelium and stroma, Bergen et al. studied single epithelial cells captured by laser microscopy [26]. Thus, Bergen et al. provided data on epithelium-specific changes in AD (larger-fold changes, see Fig. 1d–f), while our dataset also includes potential pathophysiological interactions between CP stroma and epithelium. Indeed, robust inflammatory responses were not reported by Bergen et al. [26], suggesting deductively that the immune-reactive pathophysiology occurs primarily within CP stroma.

Some limitations of our study include the small number of patients in the HuD and FTD groups, and the lack of direct confirmation of specific genes by RT-PCR. Future studies with a larger N value for HuD and FTD will increase the statistical power for disease comparisons. For post-mortem tissue, RNA stability is challenging with autopsy specimens collected at various PMIs; however, we carefully assessed RNA integrity, using only samples passing stringent criteria. Moreover, our emphasis was on significantly-enriched pathways (affecting multiple genes within a given pathway) rather than specific gene targets, with possible individual false discoveries. Any residual blood elements in specimens would unlikely explain differences in tissue mRNA among the three disease groups. The somewhat younger Ctrl group may be advantageous in avoiding potentially confounding issues with clinically-silent early dementia in an older ‘Ctrl’ cohort, otherwise presumed normal.

We used GeneGo (http://www.genego.com) and Ingenuity (http://www.ingenuity.com) sets for pathway analysis. In AD compared to Ctrls, this revealed *upregulated* inflammation genes: acute phase response, cell adhesion and cytokine, interferon, JAK-STAT signaling (for translating extracellular signals into transcriptional responses). Notable *downregulated* pathways were methionine degradation and protein translation; both are implicated in AD pathology. Claudin-5 expression was downregulated, consistent with the enhanced leakiness of the BCSFB [31] encountered in neurodegenerative diseases [26] and pathophysiology models [29]. Amyloid beta peptide (Aβ) damages CP tight junctions by activating matrix metalloproteineases, thereby increasing paracellular permeability [32]. The functional significance of such altered CP pathways, for disease outcome, awaits elucidation.

Prominent in AD was *upregulated* inflammation-related signaling. These inflammation signatures differ from cortex-associated microglial infiltration [33, 34]. Key marker genes of cortical inflammation-APOE, TREM2, TYROBP-did not upregulate in AD CP. Rather, acute phase response genes dominated the upregulation: multiple cytokine and interferon receptors, JAK-STAT signaling components, MAPK, NFκB signaling and cell adhesion. Cytokines and growth factors in disease-associated reactions in BCSFB come from brain [35], blood, or CP itself. The CP responds biochemically and transcriptionally to circulating cytokines, central injury and systemic diseases [18, 36, 37]. Localized CP immunoreactions (e.g., inflammation-resolving leukocyte trafficking) may benefit brain by sensing ‘injury signals’ flowing from brain to CSF to CP, then feeding back to make homeostatic neural adjustments [35].

At certain stages of advancing neuroinflammation (caused by brain-residing pro-inflammatory microglial responses to Aβ loads), the CP receives plasma interferon-γ as a signal to promote homeostatic transport of anti-inflammatory monocyte-derived macrophages into CSF for resolving parenchymal inflammation [12]. Disease-induced disruption of this neuroimmune interferon adjustment at the BCSFB [38] could compromise the ability of the CSF–brain to thwart AD exacerbation. The CP competently adapts to AD stress [39] by maintaining an immunosuppressant profile of factors, e.g., VEGF and TGFβ1 in CSF, to help manage brain inflammation after neuronal injury [40].

Increased expression of LRP-1, a choroidal Aβ transporter, agrees with mouse AD modeling [24]. Upregulated LRP-1 in the apical membrane expedites Aβ removal from CSF [24]. Augmented reabsorptive clearance of Aβ at the BCSFB aids the CNS because cerebral capillaries in AD extrude less Aβ [41]. This compensatory Aβ removal by CP counters the disabled microvessels [42]. Titers of inflammatory cytokines and choroidal proteins, in CSF and blood, present in different degrees in AD [43]. Activated astrocytes and microglia congregate in Aβ plaques [44]. The manner in which CP inflammatory-signaling molecules modify AD pathogenesis is heterogeneous. Acute inflammation may beneficially promote CSF clearance of affected cells and Aβ aggregates, protecting neurons. However, persistently-elevated CP–CSF cytokines and sustained activation of microglia adversely affect neurons. An effective CP will balance the beneficial vs. detrimental effects of CSF cytokine changes in AD, FTD and HuD.

Amyloid beta induces cytokine production; and astrocytes activated by Aβ, release inflammatory factors that sustain Aβ production. Clearly the CP–CSF, using soluble signals and upregulated cellular adhesion factors, appropriately distributes certain T cell phenotypes to CSF [12]; such leukocyte penetration into CSF helps to control neuroinflammation and Aβ levels in AD brain. The dynamic relationship between pro-inflammatory and anti-inflammatory cytokines in CP–CSF impacts neuroinflammation processes and AD pathology.

Peroxisome-proliferator-activated receptor (PPAR) signaling genes, including PPARδ and its obligate heterodimer RXRα, were enriched in AD CP. PPAR/RXRs are neuroprotective in AD and Aβ therapies due to anti-inflammatory and endothelial actions [43]. PPAR activation, through endogenous or synthetic ligands, likely protects CP by increasing antioxidant capacity and improving energy supply; this maintains fuel for the Na pump [45] and CSF secretion [46], and increases expression of Aβ transporters [47]. The novel GFT1803 agent (a pan-PPAR agonist that activates all 3 PPAR isoforms) attenuates Aβ loading-induced damage and neuroinflammation [48]. PPAR thus deserves attention as a potential pathway for restoring CP–CSF integrity in AD in order to counter neurodegeneration.

Significant expression differences were also observed between AD and FTD + HuD. The VEGF signaling pathway (including VEGFA and VEGF receptors FLT1 and FLT4) displayed significant upregulation in AD but not in FTD or HuD. This agrees with our previous findings of increased VEGF within AD CP [1]. VEGF is required for maintaining endothelial cell fenestration in CP capillaries [49], an important microstructural feature for delivering plasma substances into the choroidal interstitium for epithelial processing.

Cadherin, on the other hand, was upregulated in FTD and HuD but not AD. Cadherin is a superfamily of cellular adhesion molecules (CAM), that maintain tissue structure and boundaries between cells and organelles. CAM binding also modifies gene expression. Cell–cell adhesions mediate specific immune actions [50], of which there is a plethora in CP of FTD and HuD patient specimens. A cadherin family member prominent in CP is γ-protocadherin (γ-Pcdh), expressed at the apical membrane [51]. Mutation of γ-Pcdh causes ventricular collapse. Keep et al. proposed an immune and CSF dynamics role for CP γ-Pcdh [52], that co-expresses with the NaBCN2 Na transporter supporting CSF secretion. This gene may function in CP ion transport-CSF formation by way of apical–microskeletal membrane interactions with NaBCN2 that regulate ion trafficking [32]. Moreover, Kolmer immune cells, attached to CP apical surface [53], may have an altered function in neurodegenerative diseases when cadherin is upregulated.

We hypothesize that the downregulated expression observed in this study reflects failing metabolic pathways involved in choroid cell and CSF homeostasis. Reduction in methionine-degradation genes is intriguing given that excessive homocysteine, a product of methionine metabolism, is a risk factor for AD [54–57]. Methionine loading increases brain homocysteine, Aβ and phospho-tau in mouse models [58]. Decreased expression in AD CP of the methionine-degrading gene may relate to elevated homocysteine levels in CSF [59]. The impact of augmented CSF homocysteine on raising brain Aβ and tau hints that additional methionine gene studies on CP transcription factors and metabolism in neurodegenerative diseases are needed.

We also determined a decreased expression of protein translation genes, including multiple eukaryotic translation initiation factors (EIF genes) and ribosomal proteins. CP has a major role in producing and secreting CSF proteins, e.g., transthyretin that stabilizes Aβ conformation. In AD there is decreased choroidal synthesis of transthyretin [24], lowering its CSF concentration [60]. Moreover, the heat stress glucose regulatory proteins 78 and 94 in human AD CP are diminished [39], implicating suboptimal glucose or calcium homeostasis. Altered heat stress proteins at the AD BCSFB deserve examination for impact on cerebral metabolism.

Expression of mTOR associates with controlling cell growth and proliferation [61], possibly a factor as damaged choroid epithelial cells need replacement. Our finding of increased fatty acid oxidation and upstream mTOR signaling (Fig. 2), with juxtaposed downregulated protein translation, fits existing concepts suggesting altered energy metabolism in AD onset and progression. While increased PPAR activity downstream of mTOR fits the compensatory adaptation to retain CP resiliency, there is a disconnect between upregulated mTOR and the downregulated protein translation machinery typically induced by mTOR. This suggests a break in normal mTOR signaling (see Fig. 2) that could undermine CP function or resiliency when challenged with neurodegeneration. This is significant because of CP’s pivotal role in providing brain with supportive factors and immune cells that migrate across BCSFB into CSF–brain. Studies need to assess the role of the dynamic CP transcriptome in providing resiliency to the BCSFB, in order to retain CSF homeostatic reserve for staving off neurodegeneration.

## Conclusions

The AD transcript findings reported herein for bulk CP tissue compare favorably with and expand prior analysis in laser-captured epithelial cells [26]. Such concurrence is remarkable given the different tissue sampling, measurement platforms and patient cohorts. Highlights of our investigation include *upregulated* genes linked to inflammation and interferon neuroimmune homeostasis, as well as to JAK-STAT and mTOR; and *downregulated* genes for methionine degradation, protein translation and claudin-5 (tight junction). CP is a complex homeostatic tissue. The BCSFB undergoes deleterious, but sometimes functional and adaptive, changes in dementia-related pathophysiology. The enriched JAK-STAT and mTOR pathways (Fig. 2), respectively, are likely instrumental in promoting adaptive transcriptional responses and epithelial repair/replacement when CP is harmed by injuries associating with neurodegeneration. Analyzing biological pathway mechanisms expedites specific pharmacologic targeting.

Future transcriptome work with larger cohorts should delineate gene expression by demographic endpoints, Braak staging, Aβ plaque score, disease duration, ApoE genotype, co-morbidity, and other disease characteristics. The transcriptome distinctions here precisely describe CP–CSF function in, and response to, certain neuropathologies: AD vs. FTD vs. HuD. This categorical approach provides crucial knowledge on the BCSFB role in pathogenesis; and hopefully should improve prophylaxis of various neural diseases. The goal: To identify exact CP targets to exploit when implementing pharmacologic/genetic therapies to alleviate CSF–brain metabolic distortions in dementia.

## Additional files

**Additional file 1: Table S1.** Sample Annotations and Patient Demographics: ‘QC passed’ indicates samples passing both RNA and Affymetrix quality control, and used for analysis. APOE; genotype data consist of combinations of the ε2, ε3 and ε4 alleles, with ε4 carrying the highest risk for brain injury; Bioanalyzer (BA); RNA Integrity (RIN).

**Additional file 2: Table S2.** T-test results for choroid plexus RNA from 6 control, 7 Alzheimer’s disease, 4 FTD and 3HUD subjects: T-test p-values and expression differences (Log_10_ Disease/Ctrl) for each disease type are provided for all 5760 Affymetrix probesets measured. Values for the AD vs. HuD + FTD subject comparison are also provided.

**Additional file 3: Table S3.** Geneset Annotations: Worksheets contain pathways enriched among genes upregulated in AD vs. Ctrl (S3a); downregulated in AD vs. Ctrl (S3b); upregulated in AD but not in HuD + FTD vs. Ctrl (S3c); and upregulated in HuD + FTD but not AD vs. Ctrl (S3d). Hypergeometric p-values (p-value), Bonferroni-corrected p-values (E-value), overlaps and input and background set sizes are provided.

**Additional file 4: Table S4.** The top 10 most upregulated and top 10 most downregulated genes in the AD vs. Ctrl comparison, with p < 0.01. Multiple probe set data, where available, are included in the comparisons.

## Abbreviations

Aβ: amyloid beta
AD: Alzheimer’s disease
APOE: apolipoprotein
BCSFB: blood–CSF barrier
CP: choroid plexus
CSF: cerebrospinal
EIF: eukaryotic translation initiation factors
FDR: false discovery rate
FTD: frontotemporal dementia
HuD: Huntington’s disease
JAK-STAT: Janus kinase/signal transducers and activators of transcription
LRP1: low density lipoprotein receptor-related protein 1
mTOR: mechanistic target of rapamycin
PMI: post-mortem interval
PPAR: peroxisome-proliferator-activate receptor
RAGE: receptor for advanced glycation end product
TGFβ: transforming growth factor beta
VEGF: vascular endothelial growth factor
